# Junctionless ferroelectric field effect transistors based on ultrathin silicon nanomembranes

**DOI:** 10.1186/1556-276X-9-695

**Published:** 2014-12-23

**Authors:** Ronggen Cao, Gaoshan Huang, Zengfeng Di, Guodong Zhu, Yongfeng Mei

**Affiliations:** Department of Materials Science, Fudan University, Shanghai, 200433 People’s Republic of China; State Key Laboratory of Functional Materials for Informatics, Shanghai Institute of Microsystem and Information Technology, Chinese Academy of Sciences, Shanghai, 200050 People’s Republic of China

**Keywords:** Silicon nanomembrane, Ferroelectric polymer, Ferroelectric field effect transistor, Junctionless

## Abstract

The paper reported the fabrication and operation of nonvolatile ferroelectric field effect transistors (FeFETs) with a top gate and top contact structure. Ultrathin Si nanomembranes without source and drain doping were used as the semiconducting layers whose electrical performance was modulated by the polarization of the ferroelectric poly(vinylidene fluoride trifluoroethylene) [P(VDF-TrFE)] thin layer. FeFET devices exhibit both typical output property and obvious bistable operation. The hysteretic transfer characteristic was attributed to the electrical polarization of the ferroelectric layer which could be switched by a high enough gate voltage. FeFET devices demonstrated good memory performance and were expected to be used in both low power integrated circuit and flexible electronics.

## Background

In the past few years, with the development of silicon-on-insulator (SOI) process techniques [1], Si nanomembranes (SiNMs) have attracted much attention due to their unique properties, such as piezoelectric effect and high speed carrier mobility, and thereof potential applications in flexible electronics [2–6]. SiNM-based devices can be built on one or both sides, which are more immune to short-channel effects and have advantages such as faster and lower voltage/power operation and the compatible manufacturing process with current integrated circuit [7–11]. As we know, nonvolatile memories are a kind of critical microelectronic devices, among which ferroelectric memories have shown large potential especially in flexible nonvolatile memories based on ferroelectric polymer and oxide [12] or organic [13] semiconductors. However, till now, few works have been reported on SiNM-based nonvolatile memories, though such devices are expected to effectively reduce device dimensions, catch up with modern integrated circuit process, and overcome the obstacle in fabricating an ultrashallow junction for ‘gated resistors’ [14, 15]. Here, we report the feasibility and operation of SiNM-based ferroelectric field effect transistor (FeFET) memories.

## Methods

**Figure 1:**
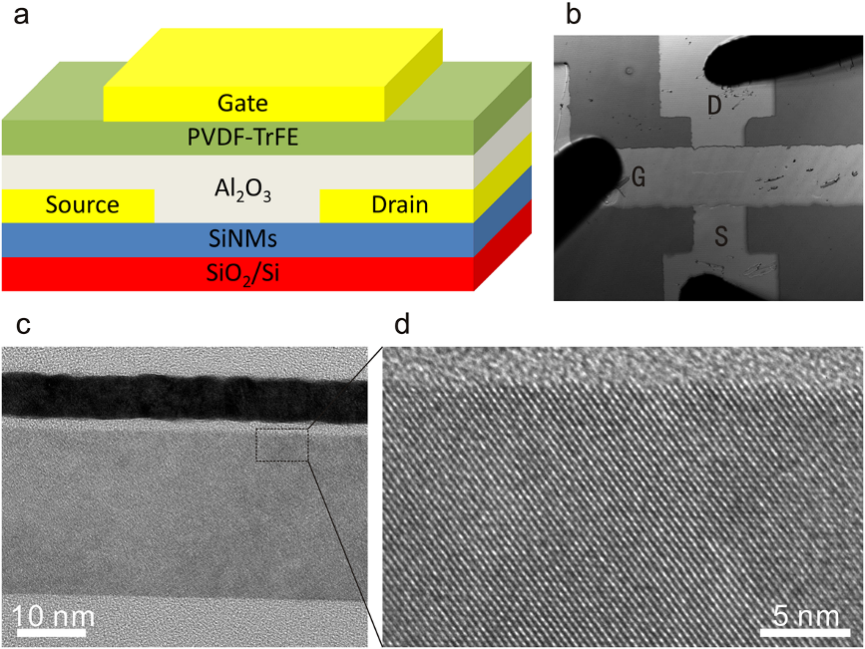
**Schematic, optical microscope image, and TEM cross-section images. (a)** Schematic of SiNM-based FeFET devices, **(b)** Optical microscope image of the electrical measurements by probe method, and **(c, d)** TEM cross-section images of SiNMs.

The device structure is shown as the inset in Figure 1a. The original SiNMs with a boron doping level of 10^15^ cm^-3^ (part of SOI wafer with Si/SiO_2_ thickness of 50/150 nm) were bought from SOITEC Inc. (Bernin, Isère, France), and the TEM cross-section images of SiNM are shown in Figure 1c,d. Al electrodes (100 nm thick) were first deposited onto SiNMs by electron beam evaporation with a hard mask to form source and drain patterns with a channel length of 80 μm and a width of 1 mm. The source and drain were not further implanted. Then, a 10-nm thick Al_2_O_3_ buffer layer was deposited by atomic layer deposition. Ferroelectric poly (vinylidene fluoride trifluoroethylene) [P(VDF-TrFE)] copolymer films with VDF/TrFE molar ratio of 77/23 were spin-coated onto the Al_2_O_3_ layer and then annealed at 138°C for 5 h to increase their degree of crystallinity. The thickness of annealed ferroelectric films was about 100 nm, determined by a scanning probe microscope (UltraObjective, Surface Imaging Systems, Herzogenrath, Germany). Finally, 100-nm thick Al electrodes were thermally evaporated to form the gate electrode. Electrical measurements were performed in a dark environment by probe method with Keithley 4200 semiconductor parameter analyzer (Keithley Instruments Inc., Cleveland, Ohio, USA), as shown in Figure 1b. During all electrical measurements, the source electrode was electrically grounded.

## Results and discussion

**Figure 2:**
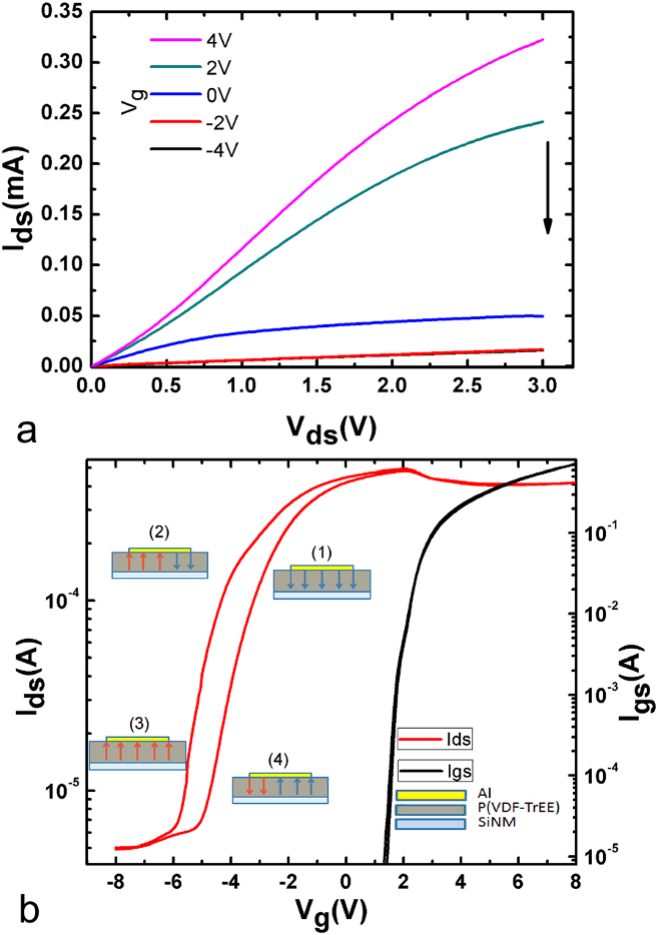
**Output and transfer and leakage characteristics SiNM-based FeFETs. (a)** Output and **(b)** transfer and leakage characteristics. Insets show the schematic diagram of operation mechanism.

The output characteristics of the SiNM-based FeFETs are shown in Figure 2a. The source-drain voltage (*V*_ds_) was swept from 0 to 3 V, while the gate voltage (*V*_g_) changed between +4 and -4 V. A typical output characteristic of SiNM-based field effect transistors is observed. The source-drain current (*I*_ds_) is hard to be saturated at positive *V*_g_, though the maximum *V*_ds_ is set to 3 V. This should be due to the fact that the substrate is not electrically grounded and the potential of the SiNMs increases when the current flows through the PN junction of the drain, causing the increase of the channel conductance.

Transfer characteristics of our FeFETs were determined by sweeping *V*_g_ between ±8 V at a constant *V*_ds_ of 0.5 V. To well-illuminate the experimental results, we define two *V*_g_ scanning directions: forward scan corresponds to *V*_g_ sweeping from negative to positive voltage, while backward scan corresponds to *V*_g_ from positive to negative voltage. Different from the typical metal-oxide-silicon field effect transistors, in which both transfer curves from the forward and the backward scans follow nearly the same trace, the FeFETs show significant hysteresis during transfer measurements (Figure 2b) due to the insertion of the ferroelectric P(VDF-TrFE) film between the gate and the oxide layers. The transfer loop in Figure 2b shows the device’s on/off ratio of about 10^2^ and the width of memory window of 0.75 V, which is defined as the gap of *V*_g_ when *I*_ds_ is half of its maximum value in a complete hysteresis loop. Furthermore, when the gate voltage is lower than 2.0 V, the gate leakage current *I*_gs_ is on the order of 10^-8^ A, about 2 orders of magnitude lower than *I*_ds_. During the electrical measurements by probe method, the mechanical stress applied by the probes causes the compression of the insulating layers between gate and source/drain electrodes and thus decreased film thickness results in the increased leakage current *I*_gs_ between gate and source, as is also shown in the leakage current curve of Figure 2b. With the further increase of *V*_g_ from 2 to 8 V, the leakage current quickly increases from 10 nA to 0.7 mA. The increased leakage current partly counteracts the further increase of *I*_ds_ especially at a gate voltage larger than 2 V and thus results in the decrease of *I*_ds_ with further increased gate voltage.

Note that both output and transfer characteristics indicate our FeFETs have a typical n-channel depletion mode (NNN), though the device is based on p-doped silicon without special source and drain doping. Here, the n-channel depletion mode is due to aluminum-silicon interaction. The work function of aluminum and electron affinity of silicon are 4.2 and 4.01 eV, respectively. At the Al/Si interface, the separation between the Fermi level and conduct band is only 0.27 eV (<1.12 eV/2), resulting in the change of the type of the silicon to n-type near the interface. At the same time, the channel is changed to n-type by fixed charges in the gate oxide. The same experimental observation was also reported in a similar Al/Si device structure [5].

The insets in Figure 2b schematically explain the origin of the electrical hysteresis (i.e., memory window) induced by the bistable orientation of electrical dipoles in the ferroelectric layer. These well-oriented dipoles induce a built-in voltage (*V*_in_) which causes the shift of the threshold voltage (*V*_th_) in the semiconducting layer [12]. Note that voltage drop on the ferroelectric layer larger than the coercive voltage (approximately 4.8 V) can lead to re-orientation of the electrical dipoles. During the backward scan, the initial applied gate voltage of +8 V is high enough to cause polarization reversal in the ferroelectric layer with electrical dipoles aligning downwards to the SiNM (inset 1), which contributes positive *V*_in_ to the SiNM layer and thus results in a *V*_th_ shift toward the negative voltage. On the other hand, during the forward scan, the initial applied voltage of -8 V induces the re-orientation of the dipoles aligning against the SiNM layer (inset 3), causing a *V*_th_ shift to the positive voltage. The insets 2 and 4 schematically show the orientation of the electrical dipoles during *V*_g_ sweeping, which correspondingly causes the tuning of *V*_in_ and then *V*_th_. As a result, a hysteresis loop can be expected as shown in Figure 2b.

**Figure 3:**
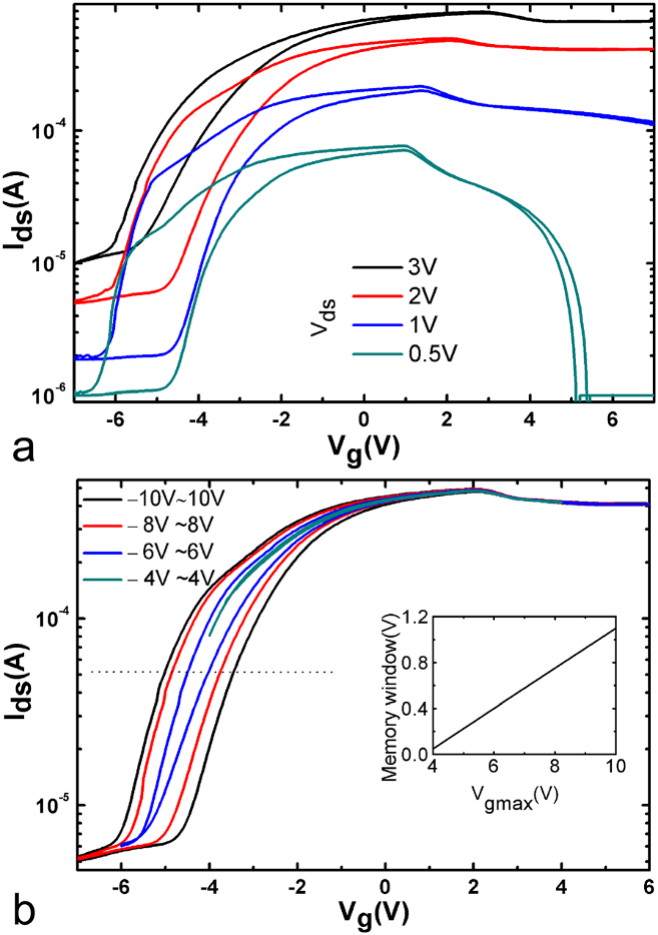
**Drain and gate voltage dependence of the transfer characteristics of SiNM-based FeFETs. (a)** Drain and **(b)** gate voltage dependence of the transfer characteristics. Inset shows the dependence of the width of memory window on *V*_gmax_.

To present a complete view of the electrical properties in the current devices, we also measured the transfer characteristics at various *V*_ds_ and noticed that *V*_ds_ had significant influence on the memory window, especially the device’s on/off ratio. The change of the transfer loops with *V*_ds_ is shown in Figure 3a, where gate voltage was swept between ±8 V. The width of memory windows almost remains constant at about 0.75 V, regardless of *V*_ds_ values. However, the device’s on/off ratio reduces significantly from 10^2^ to 10^1^ with the decrease of *V*_ds_ from 3 to 0.5 V.

Gate voltage determines the polarization in the ferroelectric layer and thus influences the memory window. To explore the mechanism behind this, we carried out more electrical characterizations on our devices. We determined the influence of *V*_gmax_ on the memory window, where *V*_gmax_ was the applied maximum gate voltage during one measurement of a whole hysteresis loop. Typical results are shown in Figure 3b, where *V*_ds_ was fixed at 3 V and *V*_g_ was swept between ± *V*_gmax_. Obviously, the width of the memory window increases with *V*_gmax_, and the device’s on/off ratio shows negligible change when *V*_gmax_ is larger than 6 V. The inset in Figure 3b demonstrates the relationship between window width and *V*_gmax_: in our experimental condition, window width increases linearly from 0.05 to 1.1 V with the increase of *V*_gmax_ from 4 to 10 V, indicating more dipole switching and thus larger *V*_in_ with the increase of *V*_gmax_.

**Figure 4:**
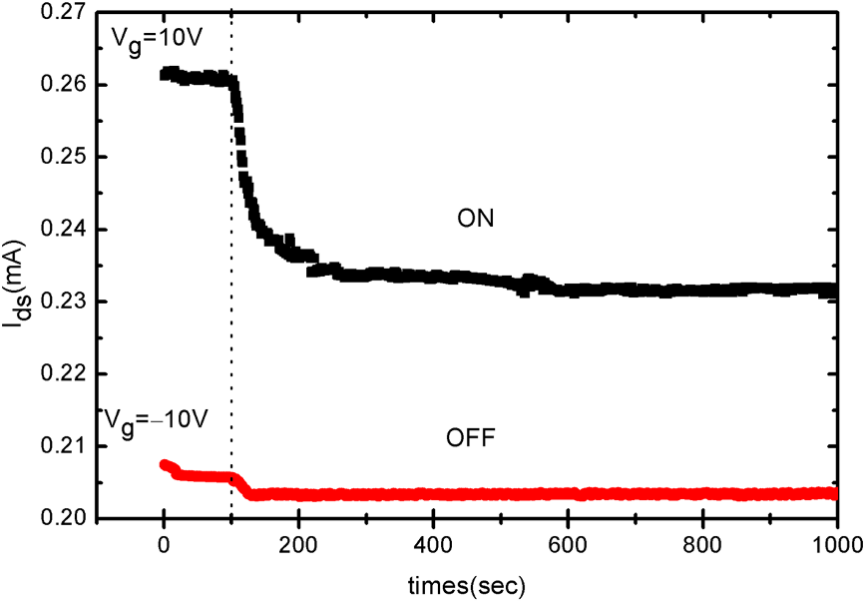
**Retention characteristic of the SiNM-based FeFETs.** ON and OFF states were written by 100-s pulses with amplitudes of 10 and -10 V, respectively.

Retention performance is especially important for nonvolatile memories, which determines the lifetime of the recorded data. Usually, as for nonvolatile memories, retention characterization should be conducted at 0-V gate voltage to meet the nonvolatile requirement. So here, the retention characteristic of SiNM-based FeFETs was measured by first applying writing gate pulses with a duration of 100 s and amplitude of 10 and -10 V and then recording *I*_ds_ at *V*_g_ = 0 V and *V*_ds_ = 1 V, respectively, at preset time points. Typical results are shown in Figure 4, where *I*_ds_ values in both ON and OFF states are plotted as a function of time. Here the ON state corresponds to that written by the +10-V gate pulse while the OFF state to the -10-V pulse. During the writing processes, ON and OFF state currents keep constant at 0.26 and 0.206 mA, respectively. Once the gate pulse is removed, the ON state *I*_ds_ sharply decreases to 0.23 mA within 130 s and then keeps nearly unchanged in the following 770 s. This sharp decrease of ON state current may be attributed to the depolarization in the ferroelectric layer due to the lack of charge compensation during the application of positive gate voltage, which is considered as one of the main causes of the worse retention performance in ferroelectric field effect transistors [16, 17]. On the other hand, the OFF state *I*_ds_ slightly decreases to 0.203 mA after the removal of the gate pulse and then keeps constant. In the whole retention measurement, the separation between ON and OFF state current decreases from 54 to 27 μA and the ON and OFF states can still be well distinguished. Especially after the sharp decrease of ON state current in the initial 130 s, both ON and OFF states maintain their currents well, indicating that the SiNM-based FeFETs exhibit good retention performance.

Note that, in our measurements of transfer characteristic, the whole hysteretic loops shift to the negative gate voltage, as shown in Figures 2b and 3. Such a shift is not due to the built-in voltage caused by the orientation of electrical dipoles in the ferroelectric layer, but due to space charges trapped in the ferroelectric layer and/or the interface between the ferroelectric and its adjacent layers, i.e., imprint effect [18], which is actually quite common in ferroelectric films and devices [19]. Nevertheless, this shift reduces the memory window measured at *V*_g_ = 0 V, resulting in a low on/off ratio of only 1.14 in the retention measurements in Figure 4. In fact, as for the transfer loop shown in Figure 2b, the maximum on/off ratio of 6.3 occurs at a *V*_g_ of -4.8 V, while the maximum separation of 0.11 mA between the ON and OFF state *I*_ds_ values occurs at a *V*_g_ of -3.6 V. To get even better memory performance especially at a *V*_g_ of 0 V, further measures should be taken to inhibit space-charge-induced shift in transfer measurements.

Although the SiNM-based FeFET device has been fabricated with good memory performance, the device needs to be further optimized. First, compared with the bulk Si, SiNM with a low doping concentration provides fewer carriers to be modulated by the ferroelectric layer, resulting in a lower switching ratio. In order to achieve good FET characteristics, SiNMs should be heavily doped [20]. Second, SiNMs should be even thinner to obtain a high on/off ratio due to easier gate control [21]. Third, SiNMs can be transferred to flexible substrates and thus flexible ‘junctionless’ FeFETs can be expected [22].

## Conclusions

In summary, nonvolatile SiNM-based FeFETs have been fabricated by integrating ferroelectric polymer thin films and ultrathin SiNMs. Electrical characterizations show that such devices have hysteretic transfer characteristic due to the modulation of electrical polarization in the ferroelectric layer. The devices show good memory performance with the device’s on/off ratio up to 10^2^ and memory window width as high as 1.1 V. Such SiNM-based FeFETs exhibit good retention performance and are expected to be used in low power integrated circuit and flexible electronics.
